# Zinc supplementation in pre-diabetes: study protocol for a randomized controlled trial

**DOI:** 10.1186/1745-6215-14-52

**Published:** 2013-02-19

**Authors:** Priyanga Ranasinghe, Ranil Jayawardena, ASAD Pigera, Prasad Katulanda, Godwin R Constantine, Priyadarshani Galappaththy

**Affiliations:** 1 Department of Pharmacology, Faculty of Medicine, University of Colombo, Colombo, Sri Lanka; 2 Institute of Health and Biomedical Innovation, Queensland University of Technology, Brisbane, QLD, Australia; 3 Department of Clinical Medicine, Faculty of Medicine, University of Colombo, Colombo, Sri Lanka

**Keywords:** Zinc supplementation, Pre-diabetes, Sri Lanka, Adults

## Abstract

**Background:**

The number of people with diabetes is increasing worldwide, especially in developing South Asian countries. Therefore, preventing diabetes at the early stages has become an important issue. Recent clinical trials, systematic reviews, and meta-analysis have shown that zinc has beneficial effects on glycemic and metabolic control in those with diabetes. The present study is designed to evaluate the effects of zinc supplementation on glycemic control and other metabolic parameters in those with pre-diabetes.

**Methods/design:**

The study will be conducted as a randomized, double-blind, placebo-controlled clinical trial for a period of 12 months at the Faculty of Medicine, University of Colombo, Sri Lanka. The study has been approved by the Ethics Review Committee of Faculty of Medicine, University of Colombo (EC/11/189). A total of 200 adults (age 18–60 years) with pre-diabetes will be recruited for the study. They will be stratified according to age, gender, and body mass index and randomly assigned into the test and placebo groups on a 1:1 ratio. The zinc capsules, each weighing 456 mg, will contain the following ingredients:zinc sulfate monohydrate 55.096 mg (elemental zinc 20 mg), lactose monohydrate 399.504 mg, and stearic acid 1.400 mg. The placebo capsule with the same weight will be comprised of lactose monohydrate 454.600 mg and stearic acid 1.400 mg. The subjects will receive either zinc 20 mg capsules or placebo daily for a period of 12 months. The study drugs will be double blinded to both investigators and subjects. The visits and the evaluations will be done as follows: screening (visit 0), 1 month (visit 1), 3 month (visit 2), 6 month (visit 3), and 12 month (visit 4). The following primary outcome measures will be evaluated:fasting plasma glucose (FPG), post oral glucose tolerance test (OGTT), serum insulin, HbA1c, total/low-density lipoprotein (LDL)/high-density lipoprotein (HDL) cholesterol, triglycerides, serum zinc, and appetite using a visual analog scale. Secondary outcome measures include: blood pressure, anthropometry, and dietary assessment using a validated food frequency questionnaire. Data will be analyzed using SPSS v16.

**Discussion:**

The present protocol will aim to establish the beneficial effects of zinc supplementation on disease progression in those with pre-diabetes and also establish its effectiveness in the prevention of diabetes mellitus.

**Trial registration:**

Sri Lanka Clinical Trial Registry: SLCTR/2012/010

## Background

The prevalence of diabetes and pre-diabetes is increasing worldwide because of population growth, aging, urbanization, and increasing prevalence of obesity and physical inactivity. The South Asian region is currently experiencing an epidemic of diabetes and its associated complications [1,2]. The projected increase in prevalence of diabetes is out of proportion to the estimated increase in population [3]. In 1990, the prevalence of diabetes in Sri Lanka was 2.5%; however, most recent studies show more than 1/5 of Sri Lankan adults are dysglycemic [4,5]. The prevalence of diabetes and pre-diabetes in the Sri Lankan adult population in 2005–2006 was 10.3% and 11.5% respectively [5]. Studies from developed countries have shown that the direct cost involved in the treatment of diabetes and its associated complications are high [6]. Furthermore the indirect costs due to early mortality, morbidity, and loss of productivity are likely to be much higher. This is even more pronounced in developing countries of the South Asian region, which are faced with the highest burden of disease [1]. Therefore, simple and effective preventive measures for those at risk of developing diabetes are a timely requirement.

Insulin is stored in β-cells of the pancreas in granules that contains a variable number of zinc atoms. Studies have shown that zinc plays an important role in insulin synthesis, storage, secretion, and action, while also being involved in various stages of carbohydrate and protein metabolism [7,8]. Oxidative stress plays an important role in the pathogenesis of diabetes and its complications. Impaired synthesis of antioxidant enzymes, such as superoxide dismutase and glutathione peroxidase, may be associated with increased oxidative stress [9]. Zinc is an integral structural component of these antioxidant enzymes, and zinc deficiency may impair their synthesis. *In-vitro* studies in animal models and in humans have demonstrated that zinc supplementation improves glycemic control and other metabolic parameters in diabetes [10-13]. A recent systematic review and meta-analysis on the effects of zinc supplementation in patients with diabetes showed that zinc supplementation reduces blood glucose, total cholesterol, and LDL cholesterol while improving glycemic control as demonstrated by a reduction in HbA1c [14]. Zinc supplementation has also shown beneficial effects on insulin resistance and components of the metabolic syndrome in pre-pubertal obese children [15]. However, at present there are no studies evaluating the long-term effects of zinc supplementation in prevention of disease in those at risk of diabetes. The present study aims to evaluate the effects of zinc supplementation on the progression of disease in patients with pre-diabetes from Sri Lanka and determine the metabolic effects of zinc supplementation on glycemic control. In addition, we also aim to evaluate effect of zinc supplementation on body weight and appetite in those with pre-diabetes.

## Methods/design

### Objectives and hypothesis

Objectives: The study aims to evaluate the effects of zinc supplementation on the progression of disease in patients with pre-diabetes from Sri Lanka and determine the metabolic effects of zinc supplementation on glycemic control. Furthermore, we aim to evaluate the effects of zinc supplementation on appetite and body weight in patients with pre-diabetes.

Hypothesis: The glucose concentration of pre-diabetic patients who are treated with elemental zinc 20 mg daily will be lower than that of the control group. We also hypothesize that other diabetes-related metabolic parameters (serum insulin, total cholesterol, triglyceride, HDL cholesterol, LDL cholesterol, and blood pressure) will be improved in the treatment group in comparison to the control group.

### Study design

**Figure 1:**
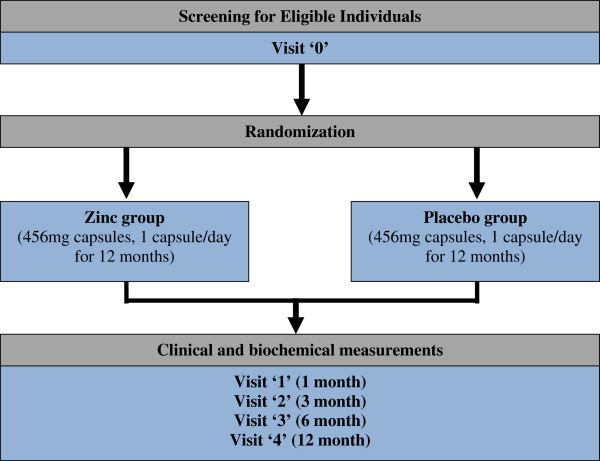
Schematic representation of study design.

The study will be conducted as a randomized, double-blind, placebo-controlled clinical trial for a period of 12 months to assess the efficacy of daily zinc 20 mg supplementation in pre-diabetes. The study will be conducted at the Faculty of Medicine, University of Colombo, Sri Lanka. Figure 1 provides an overview of the study.

### Sample size

The number of patients required for determination of a 20% reduction of fasting plasma glucose in the treatment arm in comparison with the placebo arm at 90% power and 95% confidence interval with a dropout rate of 30% is 100 patients per arm. Hence, a total of 200 subjects with pre-diabetes will be recruited for the study.

### Population

Pre-diabetes is defined as the presence of fasting plasma glucose levels between 110–125 mg/dl or 2-hpost-oral glucose plasma glucose levels between 140–199 mg/dl or both [World Health Organisation (WHO) 2006 criteria] [16]. Study participants will be recruited for the study after obtaining informed written consent.

### Inclusion and exclusion criteria

#### Inclusion criteria

•Both genders between the ages of 18–60 years, eligible for study through a screening test confirming the presence of pre-diabetes as defined above.

#### Exclusion criteria

•On any other vitamin or mineral supplementations or the current use of a weight loss medicine or dietary modification.

•History of diabetes mellitus or any metabolic disease.

•Alcohol consumption > 20 g/day.

•Presently having acute diseases or other untreated illness requiring treatment.

•Impaired hepatic or renal functions.

•Lactation, pregnancy or unwillingness to use an effective form of birth control for women of child-bearing years.

•History or presence of any condition, in the investigator’s opinion, that would endanger the individual’s safety or affect the study result.

#### Suspension criteria

•Subject’s demand to discontinue the study.

•Serious adverse events or unusual changes in clinical test results.

•Principal investigator’s decision to terminate the study (low rates of compliance, complications, or unable to sustain the study for various reasons).

### Randomization

Subjects will be stratified according to age, gender, and body mass index and randomly assigned into the test and placebo groups at a 1:1 ratio according to the ‘random number table’ generated by SPSS v16.0 software package (SPSS Inc., Chicago, IL, USA).

### Blinding

The study drugs are double blinded to both investigator and subject. The drug manufacturing will be done by SAP enterprises (PVT) Ltd., Colombo, Sri Lanka, and they will be responsible for the labeling of the zinc capsule and placebo with code numbers.

### Interventions

The treatment drug is a capsule containing elemental zinc 20 mg as the active ingredient; it will have a white-colored body and a cap. The constituents of the zinc capsule are summarized in Table 1. The placebo capsule will contain lactose monohydrate (454.600 mg) and stearic acid (1.400 mg). The placebo will be manufactured to have a similar appearance, shape, weight, taste, and color as thezinc 20 mg capsule. The subjects will receive either one capsule of 20 mg zinc or an identical placebo daily, taken 1 h before breakfast for a period of 12 months.

**Table 1:** Constituents of zinc capsule

	**Specification**	**Quantity per capsule**	**Status**
Zinc sulfate (ZnSO_4_) monohydrate*	USP	55.096 mg	Active ingredient
Lactose monohydrate	BP	399.504 mg	Filler
Stearic acid	USP	1.400 mg	Lubricant
Total weight		456.00 mg	

*Contains 20 mg of elemental zinc.

### Study groups

a. Treatment group: zinc capsule

b. Control group: placebo

### Study period

The study will be conducted for a period of 12 months. The visits and the evaluations will be done as follows: screening (visit 0), 1 month (visit 1), 3 month (visit 2), 6 month (visit 3), and 12 month (visit 4).

### Outcomes

Primary outcome index: The following biochemical assessments will be done at baseline, at the stated intervals, and on completion: FPG, OGTT, serum insulin, HbA1c, total cholesterol, triglycerides, LDL cholesterol, HDL cholesterol, and serum zinc. Furthermore, appetite will be evaluated using the visual analog scale (VAS).

Secondary outcome index:

a. Measurement of systolic (SBP) and diastolic blood pressure (DBP).

b. Anthropometric assessment such as body weight, height, body mass index (BMI), waist circumference (WC), hip circumference (HC), and waist: hip ratio (WHR).

c. Dietary assessment using a validated food frequency questionnaire (FFQ) [17].

The primary and secondary outcomes will be measured at the screening visit (visit 0), 1-month visit (visit 1), 3-month visit (visit 2), 6-month visit (visit 3), and 12-month visit (visit 4). The changes between visit 0 and visit 4 measurements will be analyzed for the primary outcome indexes.

Safety assessment index: The following information will be recorded/measured for the safety assessment: vital signs, general medical examinations, full blood count (FBC), renal function test, liver function test, and adverse events. Liver profile, renal profile, and FBC will be done at the screening day (visit 0), after 6 months (visit 3), and at the end of the study (visit 4). All other assessments will be done at each of the four visits.

### Procedures

#### Recruitment

Participants will be recruited by open advertisement in newspapers, online, and notice boards at hospitals. Those who see the trial poster will visit the trial site voluntarily.

#### Study schedule

The detailed items that will be measured at every visit are described in Table 2.

**Table 2:** A brief study schedule at every visit

	**Visit 0 (screening visit)**	**Visit 1 (1 month)**	**Visit 2 (3 month)**	**Visit 3 (6 month)**	**Visit 4 (12 month)**
Informed consent form	•				
Recording demographic data	•				
Medical history taking	•				
Physical examination^1^	•	•	•	•	•
FPG	•	•	•	•	•
OGTT	•	•	•	•	•
Serum insulin	•	•	•	•	•
Lipid profile^2^	•	•	•	•	•
HbA1c	•	•	•	•	•
Serum zinc	•	•	•	•	•
Liver function^3^	•			•	•
Renal function^4^	•			•	•
FBC^5^	•	•	•	•	•
Blood pressure	•	•	•	•	•
Electrocardiogram	•				•
Food frequency questionnaire	•	•	•	•	•
Appetite (VAS)	•	•	•	•	•

^1^Body weight, height, waist circumference and hip circumference; ^2^total cholesterol, triglyceride, LDL cholesterol, and HDL cholesterol; ^3^AST, ALT, g-GTP, total bilirubin, albumin, and total protein; ^4^creatinine and blood urea nitrogen (BUN); ^5^WBC, RBC, hemoglobin (Hb), hematocrit, and platelet count.

### Measurement tools

#### Anthropometric measurements

Body weight will be measured using a calibrated electronic floor scale (SECA 815 by SECA GmbH & Co. Kg., Hamburg, Germany) to the nearest 0.1 kg. Height will be measured to the nearest 0.1 cm using an upright plastic portable Stadiometer (SECA 217 by SECA GmbH & Co. Kg., Hamburg, Germany). BMI will be calculated as weight (in kilograms) divided by the square of height (in meters). Waist circumference (WC) will be measured with a non-elastic tape (SECA 203 by SECA GmbH & Co. Kg., Hamburg, Germany) at a point midway between the lower border of the rib cage and the iliac crest at the end of normal expiration. Similarly, the hip circumference also will be measured at the widest part of the buttocks atthe intertrochanteric level to the nearest 0.1 cm. All anthropometric measurements will be made by using standard equipment and following WHO guidelines.

#### Dietary measurements

A culturally validated FFQ will be used to obtain habitual intake of calorie, macro- and micronutrients [17]. A VAS, 10 cm in length with words anchored at each end, expressing the most positive and the most negative rating, will be used to assess hunger, satiety, fullness, and desire to eat.

### Compliance calculation

Subjects are asked to return remaining drugs and their compliance will be evaluated by using the formula given below:

$$\mathrm{Compliance}\left(\%\right)=\left[\frac{\left(\mathrm{distributed}\;\mathrm{drugs}-\mathrm{remaining}\;\mathrm{drugs}\right)}{\mathrm{distributed}\;\mathrm{drugs}}\right]\times 100$$

### Statistical analysis

Parametric and nonparametric statistical tests will be applied using SPSS version 16 (SPSS Inc., Chicago, IL, USA) and Stata/SE 10.0 (StataCorp., College Station, TX, USA) for the data analysis. For each of the outcomes, multilevel regression analysis will be used to examine differences between trial arms. For binary outcomes the model will be logistic and for continuous outcomes the model will be linear regression. All analyses will follow intention-to-treat principles and a pre-specified analysis plan. Where appropriate, sensitivity analyses will be conducted (for example, control for additional covariates and bootstrapped p values for skewed outcomes). In the case of missing data values, we will apply mean imputation and regression imputation where rates are low and consider multiple imputations where they exceed 10%.

### Adverse effect evaluation

The Recommended Daily Allowance (RDA) of zinc is 11 mg in adult males and 8 mg in females [18]. In addition, it has been shown that the therapeutic window for zinc is between 9–40 mg. Hence, 20 mg of elemental zinc is a safe dose for humans. However, in the event of a probable adverse reaction, the following precautions would ensure timely identification and management of patients:

•Reporting mechanisms will be put in place to ensure direct reporting of probable adverse events to the investigator by patients (via telephone, which will be available 24 h on all days).

•During follow-up visits, probable adverse events will be noted by history and examination and investigated in detail. All adverse effects observed will be documented in the case record form (CRF) (Additional file 1).

•All serious adverse events will be reported to the Ethics Review Committee, Faculty of Medicine, University of Colombo, and the National Pharmaco-vigilance Unit of the Department of Pharmacology, Faculty of Medicine, University of Colombo.

•A Data Safety Monitoring Board (DSMB) identified by the investigators will evaluate all the adverse events at regular intervals

•Investigations including liver function, renal function, and FBC will be assessed as detailed above.

•Termination of study: In the event of major adverse effects occurring in a significant proportion of the study population, the study would be terminated pending further investigation.

### Data collection

Data collection will be performed according to the standard operating procedures by medically trained research assistants (RAs).

### Data and biological sample handling

Data will be entered by a minimum number of dedicated staff and saved in a dedicated computer with password protection. Blood samples will be stored in a secure facility with redundant measures to ensure specimens are kept in compliant conditions at all times when in storage. Storage technologies with the capability of monitoring the temperature of samples around the clock will be utilized. After each analysis has been completed and with the approval of the principal investigator, the samples stored in the storage facility may be disposed of by the sample custodian. Asample disposal sheet (SDS) will be completed and kept for further reference.

### Ethical approvals

The study has been approved by the Ethics Review Committee (ERC) of the Faculty of Medicine, University of Colombo (EC/11/189). The trial is also registered at the Sri Lanka Clinical Trials Registry (SLCTR/2012/010). The study will be conducted in compliance with the Declaration of Helsinki and the Good Clinical Practice (GCP) guidelines.

## Discussion

In this article, we present a clinical trial design to evaluate the effects of zinc supplementation in those with pre-diabetes. To our knowledge this is one of the first randomized controlled trials evaluating the effects of long-term zinc supplementation in pre-diabetes. This study will provide the necessary groundwork for future large-scale multicenter clinical trials. Given the current enthusiasm for using various dietary supplements to improve glycemic control and metabolic parameters in those with dysglycemia, properly designed scientific evaluations are a timely requirement. Furthermore, in animal models and in humans, zinc plays an important role in the regulation of appetite [19]. However, presently there are no well-designed randomized control trials to support/refute this argument. This study will also help to provide information pertaining to appetite changes resulting from zinc supplementation. Appetite regulation could be one of the probable mechanisms responsible for beneficial effects on glycemic control. The result, positive or negative, should provide a step change in the evidence guiding current and future policies regarding dietary supplementation in prevention of diabetes.

## Trial status

Patient recruitment stage.

## Abbreviations

ALT: Alanine aminotransferase;AST: Aspartate aminotransferase;BMI: Body mass index;BUN: Blood urea nitrogen;CRF: Case report form;DBP: Diastolic blood pressure;DSMB: Drug safety monitoring board;ERC: Ethics review committee;FBC: Full blood count;FFQ: Food frequency questionnaire;FPG: Fasting plasma glucose;g-GTP: Gamma-glutamyltransferase;HC: Hip circumference;HDL: High-density lipoprotein;LDL: Low-density lipoprotein;OGTT: Oral glucose tolerance test;RA: Research assistance;RBC: Red blood cell;RDA: Recommended Daily Allowance;SBP: Systolic blood pressure;SDS: Sample disposal sheet;SLCTR: Sri Lanka clinical trial registry;SOP: Standard operating procedure;AS: Visual analog scale;WBC: White blood cell;WC: Waist circumference;WHO: World Health Organization;WHR: Waist: hip ratio

## Competing interest

The authors declare that they have no competing interests.

## Authors’ contributions

PR and RJ substantially contributed to the general idea and design of the study. PR, RJ, PG, ASADP, PK, and GRC took part in designing the protocol. PR, RJ, and PG planned the data analysis. PR and ASAD drafted the manuscript. All authors have read and consented to the manuscript.

## Supplementary Material

Additional file 1 Case Record Form (CRF). Click here for file
