# Supplementary material for: Zinc supplementation in pre-diabetes: study protocol for a randomized controlled trial
Source: Trials. 2013 Feb 19;14:52. doi: 10.1186/1745-6215-14-52 (PMC3599871; doi:10.1186/1745-6215-14-52)
Supplement: Additional file 1 — Case Record Form (CRF). [file 1745-6215-14-52-S1.doc]

**Study Title**: Effects of Zinc supplementation in pre-diabetes

**Study Code:……………..**

**Protocol Version**:…….……… **Protocol Date**:…….……….

**SCREENING AND CASE REPORT FORM**

OPD/IPD No :__________________________

Patient’s Initials :__________________________

Age/DOB :__________________________

Sex :__________________________

**Investigator:** ________________________________________________

Sponsor: National Science Foundation

Contact person: Dr Priyanga Ranasinghe

Department of Pharmacology, Faculty of Medicine, University of Colombo

Mobile : 0714-039413

Email : [priyanga.ranasinghe@gmail.com](mailto:priyanga.ranasinghe@gmail.com)

**VISIT 0 – SCREENING VISIT**

**(DAY 0)**

**OPD No.______________ Patient Initials: ___________________ Date:____/___/___**

**Written informed consent obtained? Yes No**

Date of informed consent __________________________________

Age: __________ yrs Sex: __________

1. **PRESENT COMPLAINTS**

_______________________________________________________________________________________________________________________________________________________________________________________________________________

1. **MEDICAL/ SURGICAL PAST HISTORY AND CURRENT DISEASES:**

**A) Medical/ surgical past history or current disease? Yes No**

If yes, please specify: _____________________

**B) Previous and Concomitant Treatments**

1. Any treatment stopped at study entry? Yes No

If yes, please specify:___________________________________________________

1. Any treatment which will be continued during the study? Yes No

If yes, please specify:___________________________________________________

1. **GENERAL PHYSICAL EXAMINATION**

**Temperature =__________ ^0^C**

**Anemia Jaundice Liver___________ Spleen ___________**

**Any other abnormality _________________________________________________**

**Investigator’s Signature:___________________________ Date:________________**

1. **SYSTEMIC EXAMIANTION**

**Respiratory system:____________________________________________________**

**__________________________________________________________________________________________________________________________________________**

**Gastrointestinal system:________________________________________________**

**__________________________________________________________________________________________________________________________________________**

**Central Nervous system:________________________________________________**

**__________________________________________________________________________________________________________________________________________**

1. **CARDIOVASCULAR SYSTEM EXAMINATION:**

**a) Pulse Examination:**

**Rate :_________________________/ minute**

**Rhythm :___________________________________________**

**Volume :___________________________________________**

**Force :___________________________________________**

**b) Blood Pressure (Sitting position)**

**Subject has to take 30 minutes rest before recording Blood Pressure.**

**Systolic blood pressure :___________________ mm of Hg**

**Diastolic blood pressure :___________________ mm of Hg**

**Mean blood pressure :___________________ mm of Hg**

Mean blood pressure = Diastolic blood pressure + 1/3 (Systolic blood pressure – Diastolic blood pressure)

**c) Auscultation:_______________________________________________________**

**_____________________________________________________________________**

**_____________________________________________________________________**

**Investigator’s Signature:___________________________ Date:________________**

1. **ANTHROPOMETRIC MEASUREMENT**

| **SR. NO** | **PARAMETER** | **MEASUREMENT** |
| --- | --- | --- |
| 1. | Height (cm) |  |
| 2. | Body weight (kg) |  |
| 3. | Waist circumference (cm) |  |
| 4. | Hip circumference (cm) |  |

1. **ELECTROCARDIOGRAM FINDINGS**:

Heart Rate________________________________________________________

Rhythm/ Ectopics __________________________________________________

PR interval________________________________________________________

ST segment_______________________________________________________

QRS complex______________________________________________________

Other_____________________________________________________________

**Investigator’s Signature :__________________________ Date:________________**

1. **BASELINE LABORATORY INVESTIGATIONS**

| **SR No** | **PARAMETER** | **OBSERVED VALUE** | **NORMAL RANGE** |
| --- | --- | --- | --- |
| 1. | Hemoglobin |  |  |
| 2. | RBC Count |  |  |
| 3. | WBC Count |  |  |
| 4. | Differential WBC Count | N______ E______ B_____ L_____ M_____ |  |
| 5. | Platelet Count |  |  |
| 6. | LDL |  |  |
| 7. | HDL |  |  |
| 8. | Total Cholesterol |  |  |
| 9. | Triglycerides |  |  |
| 10. | Fasting Blood Glucose level |  |  |
| 11. | OGTT |  |  |
| 12. | HbA1c |  |  |
| 13. | Serum Insulin |  |  |
| 14. | Serum Zinc |  |  |
| 15. | ALT |  |  |
| 16. | AST |  |  |
| 17. | Total bilirubin |  |  |
| 18. | Creatinine |  |  |
| 19. | Pregnancy test(females) |  |  |

**Investigator’s Signature:___________________________ Date:________________**

1. **FOOD FREQUENCY QUESTIONNAIRE**

**Follow the instructions given below before filling the food frequency questionnaire.**

- - - - Try to remember **how often** and **the average amount of each food you consumed following foods** listed in the table below **during the past month** **consumed at a meal**.
- When answering each question, think of the **amount** of that food you had, even though you may rarely have consumed the food on its own.
- If you usually consume more than one helping, sum up all the helpings you had when considering the total amount of food you had for that particular meal. Keeping this in mind, choose the serving size closest to the **total amount** you consumed.
- **Indicate the frequencies you had** for each food shown on this questionnaire **on a Daily/Weekly/Monthly basis.**
- If you mention the frequency of daily consumption for a one particular food item, you do not need to mention it again as weekly or monthly basis. Eg; consume rice 3 times per day. Then no need to mention it again as weekly and monthly basis. Look at the example given below.
- When answering the first four questions, look at given pictures carefully and select the portion sizes of rice, vegetable, meat and dhal **you are usually eating**. It can be less than A/A/between A & B/B/between B & C/C/more than C.
- Similarly, you do not need to consider about the preparation methods of each food item. In the case of jak, manioc, breadfruit and potato, you can consider both (curry and boiled form) together. If you have consumed any type of green leafy vegetables, it should come under the raw of “Common Mallum”.
- **Example**

| **Code**  **No** | **Food Type** | **Portion size** | **No of units taken at a time** | **Per**  **Day** | **Per**  **Week** | **Per**  **Month** |
| --- | --- | --- | --- | --- | --- | --- |
| 100 | Unpolished Rice (Red Kakulu) | According to picture | 1 | 3 | - | - |
| 103 | Bread | 1 slice | 4 | - | 5 | - |

1.When you eat **RICE**, did you usually eat:

| **Figure A**  **A** | **Figure B** | **Figure C** |
| --- | --- | --- |

| Less than A | A | Between A & B | B | Between A & B | C | More than C |
| --- | --- | --- | --- | --- | --- | --- |

2. When you eat **VEGETABLE**, did you usually eat:

| **Figure A**  **B** | | | **Figure B** | | | **Figure C** | | |  |
| --- | --- | --- | --- | --- | --- | --- | --- | --- | --- |
| Less than A | A | Between A & B | | B | Between A & B | | C | More than C | |

3. When you eat **MEAT**, did you usually eat:

| **Figure A**  **C** | | | **Figure B** | | | **Figure C** | | |  |
| --- | --- | --- | --- | --- | --- | --- | --- | --- | --- |
| Less than A | A | Between A & B | | B | Between A & B | | C | More than C | |

4. When you eat **DHAL**, did you usually eat:

| **Figure A**  **D** | | | **Figure B** | | | **Figure C** | | |  |
| --- | --- | --- | --- | --- | --- | --- | --- | --- | --- |
| Less than A | A | Between A & B | | B | Between A & B | | C | More than C | |

**Investigator’s Signature:___________________________ Date:________________**

| **Code**  **No** | **Food Type** | | **Portion size** | **No of units taken at a time** | | **Per**  **Day** | **Per**  **Week** | **Per**  **Month** |
| --- | --- | --- | --- | --- | --- | --- | --- | --- |
| **100** | **Cereals or equivalents** | | | | | | | |
| **101** | Unpolished Rice (Red Kakulu) | According to picture **A** | | |  |  |  |  |
| **102** | Polished rice | According to picture **A** | | |  |  |  |  |
| **103** | Bread | 1 slice | | |  |  |  |  |
| **104** | String hoppers | 1 medium size | | |  |  |  |  |
| **105** | Roti | 1 medium | | |  |  |  |  |
| **106** | Pittu | 5 cm length piece | | |  |  |  |  |
| **107** | Noodles/Macaroni/Pasta | According to picture **A** | | |  |  |  |  |
| **108** | Hoppers | 1 medium | | |  |  |  |  |
| **109** | Milk rice | 1 piece | | |  |  |  |  |
| **110** | Bun & Bakery products | 1 medium | | |  |  |  |  |
| **111** | Thosai | 1 medium | | |  |  |  |  |
| **112** | Potato | 1 table spoon | | |  |  |  |  |
| **113** | Manioc | 1 medium coconut spoon | | |  |  |  |  |
| **114** | Boiled sweet potato | 1 medium coconut spoon | | |  |  |  |  |
| **115** | Jak | 1 medium coconut spoon | | |  |  |  |  |
| **116** | Breadfruit | 1 medium coconut spoon | | |  |  |  |  |
| **117** | Ash plantain | 1 table spoon | | |  |  |  |  |
| **200** | **Vegetables** | | | | | | | |
| **201** | Coconut sambol | | According to picture **B** | |  |  |  |  |
| **202** | Beans/ long beans/ wing beans curry | |  | |  |  |  |  |
| **203** | Cabbage curry | |  | |  |  |  |  |
| **204** | Pumpkin curry | |  | |  |  |  |  |
| **205** | Brinjol curry | |  | |  |  |  |  |
| **206** | Polos curry/mallum | |  | |  |  |  |  |
| **207** | Carrot curry | |  | |  |  |  |  |
| **208** | Kohila curry | |  | |  |  |  |  |
| **209** | Kehelmuwa curry | |  | |  |  |  |  |
| **Code**  No | | **Food Type** |  |  |  |  |  |  | | --- | --- | --- | --- | --- | --- | --- | | | **Portion size** | | **No of units taken at a time** | **Per**  **Day** | **Per**  **Week** | **Per**  **Month** |
| **210** | Leaks curry | |  | |  |  |  |  |
| **211** | Bittergourd curry | |  | |  |  |  |  |
| **212** | Radish/Nohkol | |  | |  |  |  |  |
| **213** | Snakegourd/Ridgegourd | |  | |  |  |  |  |
| **214** | Beetroot curry | |  | |  |  |  |  |
| **215** | Mushroom curry | |  | |  |  |  |  |
| **216** | Tomato curry | |  | |  |  |  |  |
| **217** | Cucumber/Kakiri curry | |  | |  |  |  |  |
| **218** | Ladies fingers curry | |  | |  |  |  |  |
| **219** | Common mallum | |  | |  |  |  |  |
| **220** | Vegetable soup | | 1 cup | |  |  |  |  |
| **300** | **Pulses** | | | | | | | |
| **301** | Dhal curry (thick) | | According to picture **D** |  | |  |  |  |
| **302** | Dhal curry (watery) | | According to picture **D** |  | |  |  |  |
| **303** | Soya curry | |  |  | |  |  |  |
| **304** | Chick peas | |  |  | |  |  |  |
| **305** | Green grams | |  |  | |  |  |  |
| **306** | Samaposha | |  |  | |  |  |  |
| **400** | **Meat/Poultry and meat/Poultry products** | | | | | | | |
| **401** | Chicken | | According to picture **C** |  | |  |  |  |
| **402** | Beef/Pork/Mutton | | According to picture **C** |  | |  |  |  |
| **403** | Meat balls/sausages | | 1 medium size |  | |  |  |  |
| **404** | Egg boiled/Omelet | | 1 |  | |  |  |  |
| **405** | Dry fish /Sprat | | 1 table spoon |  | |  |  |  |
| **406** | Fish | | 1 medium piece |  | |  |  |  |
| **407** | Salmon | | 1 medium piece |  | |  |  |  |
| **408** | prawn/cuttlefish/crabs | | 1 medium |  | |  |  |  |
| **Code**  No | | **Food Type** |  |  |  |  |  |  | | --- | --- | --- | --- | --- | --- | --- | | | **Portion size** | **No of units taken at a time** | | **Per**  **Day** | **Per**  **Week** | **Per**  **Month** |
| **500** | **Fruits** | | | | | | | |
| **501** | Banana | | 1 medium |  | |  |  |  |
| **502** | Papaya | | 1 piece |  | |  |  |  |
| **503** | Apple | | 1 medium |  | |  |  |  |
| **504** | Mango | | 1 medium |  | |  |  |  |
| **505** | Orange | | 1 medium |  | |  |  |  |
| **506** | Pineapple | | Medium size |  | |  |  |  |
| **507** | Guava | | 1 medium |  | |  |  |  |
| **508** | Fruit salad | | 1 cup |  | |  |  |  |
| **509** | Dried fruits-Dates/ Resins | | 1 handful |  | |  |  |  |
| **600** | **Beverages** | | | | | | | |
| **601** | Nestamalt /Viva/Milo | | 1 glass |  | |  |  |  |
| **602** | Full cream milk | | 1 glass |  | |  |  |  |
| **603** | Non fat milk | | 1 glass |  | |  |  |  |
| **604** | Fresh milk | | 1 glass |  | |  |  |  |
| **605** | Plain tea | | 1 cup |  | |  |  |  |
| **606** | Coffee | | 1 cup |  | |  |  |  |
| **607** | Fizzy drinks | | 200 ml |  | |  |  |  |
| **608** | Cordial/Smack/Colman | | 1 glass |  | |  |  |  |
| **609** | Kola kada/rice kanji | | 1 glass |  | |  |  |  |
| **700** | **Miscellaneous** | | | | | | | |
| **701** | Yoghurt | | 1 cup |  | |  |  |  |
| **702** | Ice cream | | 1 cup |  | |  |  |  |
| **703** | Curd | | 1 cup |  | |  |  |  |
| **704** | Cheese | | 1 wedge |  | |  |  |  |
| **705** | Un-sweeten Biscuits | | 1 |  | |  |  |  |
| **706** | Cream/Sweet biscuits | | 1 |  | |  |  |  |
| **707** | Cake | | 1 medium size |  | |  |  |  |
| **Code**  No | | **Food Type** |  |  |  |  |  |  | | --- | --- | --- | --- | --- | --- | --- | | | **Portion size** | **No of units taken at a time** | | **Per**  **Day** | **Per**  **Week** | **Per**  **Month** |
| **708** | Watalappam/Pudding | | 1 cup |  | |  |  |  |
| **709** | Sweets | | 1 medium |  | |  |  |  |
| **710** | Sugar | | 1 tea spoon |  | |  |  |  |
| **711** | Toffee | | 1 |  | |  |  |  |
| **712** | Chocolate | | 1 |  | |  |  |  |
| **713** | Butter/Margarine | | 1 tea spoon |  | |  |  |  |
| **714** | Jam | | 1 tea spoon |  | |  |  |  |
| **715** | Tomato sauce | | 1 table spoon |  | |  |  |  |
| **716** | Mixtures/salted nuts | | 1 table spoon |  | |  |  |  |
| **800** | **Alcohol** | | | | | | | |
| **801** | Spirit | | 25 ml |  | |  |  |  |
| **802** | Beer | | 1 pint |  | |  |  |  |

1. **VISUAL ANALOGUE SCALES (VAS)**

- How **HUNGRY** do you feel *at this moment*?

**Not at all Very**

**HUNGRY HUNGRY**

- How **FULL** do you feel *at this moment*?

**Not at all Very**

**FULL FULL**

- How **STRONG** is your desire to eat *at this moment*?

**Not at all Very**

**STRONG STRONG**

- How **MUCH FOOD** do you think you could eat *at this moment*?

**None A large**

**amount**

**Investigator’s Signature:___________________________ Date:________________**

**CHECKLIST FOR INCLUSION AND EXCLUSION**

**INCLUSION:**

**(Please Tick (√)** Yes or No)

|  | **CRITERIA** | **YES** | **NO** |
| --- | --- | --- | --- |
| 1. | Male or Female subjects between 18-60 years of age with pre-diabetes inclusive of both limits |  |  |
| 2. | Subjects willing to give written informed consent and comply with protocol |  |  |

***If answer to any of the above questions is “No” then patient should not be included.**

**EXCLUSION:**

**(Please Tick (√) Yes or No)**

|  | **CRITERIA** | **YES** | **NO** |
| --- | --- | --- | --- |
| 1. | Alcohol consumption >20 g/day, with Alcoholic Liver Disease or cirrhosis |  |  |
| 2. | Patient with any chronic illness |  |  |
| 3. | Current use of a weight loss medicine or dietary modification |  |  |
| 4. | Lactation, pregnancy or unwillingness to use an effective form of birth control for women of child bearing years. |  |  |
| 5. | On any vitamin or mineral supplementations |  |  |
| 6. | Any condition that, in the opinion of the investigator, does not justify the patient’s inclusion in the study. |  |  |

*** If answer to any of the above questions is “Yes” then patient is to be excluded.**

**If included, then Pt Serial No. assigned :_____________________________________**

**Drug administered/Drug Code :_____________________________________**

**No. of tablets given :_____________________________________**

***Give instructions to the patient about drug and dosage.***

**Date of next Visit:________________________ Day____________________________**

**Investigator’s Signature :___________________________ Date:________________**

**VISIT 1 – FIRST VISIT**

**(After 30 days therapy)**

**OPD No.______________ Patient Initials: _________________ Date:___/___/___**

1. **PRESENT COMPLAINTS**

______________________________________________________________________________________________________________________________________________________________________________________________________________

1. **GENERAL PHYSICAL EXAMINATION**

**Temperature =__________ ^0^C**

**Anemia Jaundice Liver___________ Spleen ___________**

**Any other abnormality _________________________________________________**

1. **SYSTEMIC EXAMIANTION**

**Respiratory system:____________________________________________________**

**__________________________________________________________________________________________________________________________________________**

**Gastrointestinal system:________________________________________________**

**__________________________________________________________________________________________________________________________________________**

**Central Nervous system:________________________________________________**

**__________________________________________________________________________________________________________________________________________**

1. **CARDIOVASCULAR SYSTEM EXAMINATION:**

**a) Pulse Examination:**

**Rate :___________________________________________/ minute**

**Rhythm :__________________________________________________**

**Volume :__________________________________________________**

**Force :__________________________________________________**

**b) Blood Pressure (Sitting position)**

**Subject has to take 30 minutes rest before recording Blood Pressure.**

**Systolic blood pressure (SBP) :____________________ mm of Hg**

**Diastolic blood pressure (DBP) :____________________ mm of Hg**

**Mean blood pressure (MBP) :____________________ mm of Hg**

Mean blood pressure = DBP + 1/3 (SBP – DBP)

**c) Auscultation: _______________________________________________________**

**_____________________________________________________________________**

1. **ANTHROPOMETRIC MEASUREMENT**

| **SR. NO** | **PARAMETER** | **MEASUREMENT** |
| --- | --- | --- |
| 1. | Height (cm) |  |
| 2. | Body weight (kg) |  |
| 3. | Waist circumference (cm) |  |
| 4. | Hip circumference (cm) |  |

1. **LABORATORY INVESTIGATIONS**

| **SR No** | **PARAMETER** | **OBSERVED VALUE** | **NORMAL RANGE** |
| --- | --- | --- | --- |
| 1. | Hemoglobin |  |  |
| 2. | RBC Count |  |  |
| 3. | WBC Count |  |  |
| 4. | Differential WBC Count | N______ E______ B_____ L_____ M_____ |  |
| 5. | Platelet Count |  |  |
| 6. | LDL |  |  |
| 7. | HDL |  |  |
| 8. | Total Cholesterol |  |  |
| 9. | Triglycerides |  |  |
| 10. | Fasting Blood Glucose level |  |  |
| 11. | OGTT |  |  |
| 12. | HbA1c |  |  |
| 13. | Serum Insulin |  |  |
| 14. | Serum Zinc |  |  |

1. **FOOD FREQUENCY QUESTIONNAIRE**

1.When you eat **RICE**, did you usually eat:

| **Figure A**  **A** | **Figure B** | **Figure C** |
| --- | --- | --- |

| Less than A | A | Between A & B | B | Between A & B | C | More than C |
| --- | --- | --- | --- | --- | --- | --- |

2. When you eat **VEGETABLE**, did you usually eat:

| **Figure A**  **B** | | | **Figure B** | | | **Figure C** | | |  |
| --- | --- | --- | --- | --- | --- | --- | --- | --- | --- |
| Less than A | A | Between A & B | | B | Between A & B | | C | More than C | |

3. When you eat **MEAT**, did you usually eat:

| **Figure A**  **C** | | | **Figure B** | | | **Figure C** | | |  |
| --- | --- | --- | --- | --- | --- | --- | --- | --- | --- |
| Less than A | A | Between A & B | | B | Between A & B | | C | More than C | |

4. When you eat **DHAL**, did you usually eat:

| **Figure A**  **D** | | | **Figure B** | | | **Figure C** | | |  |
| --- | --- | --- | --- | --- | --- | --- | --- | --- | --- |
| Less than A | A | Between A & B | | B | Between A & B | | C | More than C | |

| **Code**  **No** | **Food Type** | | **Portion size** | **No of units taken at a time** | | **Per**  **Day** | **Per**  **Week** | **Per**  **Month** |
| --- | --- | --- | --- | --- | --- | --- | --- | --- |
| **100** | **Cereals or equivalents** | | | | | | | |
| **101** | Unpolished Rice (Red Kakulu) | According to picture **A** | | |  |  |  |  |
| **102** | Polished rice | According to picture **A** | | |  |  |  |  |
| **103** | Bread | 1 slice | | |  |  |  |  |
| **104** | String hoppers | 1 medium size | | |  |  |  |  |
| **105** | Roti | 1 medium | | |  |  |  |  |
| **106** | Pittu | 5 cm length piece | | |  |  |  |  |
| **107** | Noodles/Macaroni/Pasta | According to picture **A** | | |  |  |  |  |
| **108** | Hoppers | 1 medium | | |  |  |  |  |
| **109** | Milk rice | 1 piece | | |  |  |  |  |
| **110** | Bun & Bakery products | 1 medium | | |  |  |  |  |
| **111** | Thosai | 1 medium | | |  |  |  |  |
| **112** | Potato | 1 table spoon | | |  |  |  |  |
| **113** | Manioc | 1 medium coconut spoon | | |  |  |  |  |
| **114** | Boiled sweet potato | 1 medium coconut spoon | | |  |  |  |  |
| **115** | Jak | 1 medium coconut spoon | | |  |  |  |  |
| **116** | Breadfruit | 1 medium coconut spoon | | |  |  |  |  |
| **117** | Ash plantain | 1 table spoon | | |  |  |  |  |
| **200** | **Vegetables** | | | | | | | |
| **201** | Coconut sambol | | According to picture **B** | |  |  |  |  |
| **202** | Beans/ long beans/ wing beans curry | |  | |  |  |  |  |
| **203** | Cabbage curry | |  | |  |  |  |  |
| **204** | Pumpkin curry | |  | |  |  |  |  |
| **205** | Brinjol curry | |  | |  |  |  |  |
| **206** | Polos curry/mallum | |  | |  |  |  |  |
| **207** | Carrot curry | |  | |  |  |  |  |
| **208** | Kohila curry | |  | |  |  |  |  |
| **209** | Kehelmuwa curry | |  | |  |  |  |  |
| **Code**  No | | **Food Type** |  |  |  |  |  |  | | --- | --- | --- | --- | --- | --- | --- | | | **Portion size** | | **No of units taken at a time** | **Per**  **Day** | **Per**  **Week** | **Per**  **Month** |
| **210** | Leaks curry | |  | |  |  |  |  |
| **211** | Bittergourd curry | |  | |  |  |  |  |
| **212** | Radish/Nohkol | |  | |  |  |  |  |
| **213** | Snakegourd/Ridgegourd | |  | |  |  |  |  |
| **214** | Beetroot curry | |  | |  |  |  |  |
| **215** | Mushroom curry | |  | |  |  |  |  |
| **216** | Tomato curry | |  | |  |  |  |  |
| **217** | Cucumber/Kakiri curry | |  | |  |  |  |  |
| **218** | Ladies fingers curry | |  | |  |  |  |  |
| **219** | Common mallum | |  | |  |  |  |  |
| **220** | Vegetable soup | | 1 cup | |  |  |  |  |
| **300** | **Pulses** | | | | | | | |
| **301** | Dhal curry (thick) | | According to picture **D** |  | |  |  |  |
| **302** | Dhal curry (watery) | | According to picture **D** |  | |  |  |  |
| **303** | Soya curry | |  |  | |  |  |  |
| **304** | Chick peas | |  |  | |  |  |  |
| **305** | Green grams | |  |  | |  |  |  |
| **306** | Samaposha | |  |  | |  |  |  |
| **400** | **Meat/Poultry and meat/Poultry products** | | | | | | | |
| **401** | Chicken | | According to picture **C** |  | |  |  |  |
| **402** | Beef/Pork/Mutton | | According to picture **C** |  | |  |  |  |
| **403** | Meat balls/sausages | | 1 medium size |  | |  |  |  |
| **404** | Egg boiled/Omelet | | 1 |  | |  |  |  |
| **405** | Dry fish /Sprat | | 1 table spoon |  | |  |  |  |
| **406** | Fish | | 1 medium piece |  | |  |  |  |
| **407** | Salmon | | 1 medium piece |  | |  |  |  |
| **408** | prawn/cuttlefish/crabs | | 1 medium |  | |  |  |  |
| **Code**  No | | **Food Type** |  |  |  |  |  |  | | --- | --- | --- | --- | --- | --- | --- | | | **Portion size** | **No of units taken at a time** | | **Per**  **Day** | **Per**  **Week** | **Per**  **Month** |
| **500** | **Fruits** | | | | | | | |
| **501** | Banana | | 1 medium |  | |  |  |  |
| **502** | Papaya | | 1 piece |  | |  |  |  |
| **503** | Apple | | 1 medium |  | |  |  |  |
| **504** | Mango | | 1 medium |  | |  |  |  |
| **505** | Orange | | 1 medium |  | |  |  |  |
| **506** | Pineapple | | Medium size |  | |  |  |  |
| **507** | Guava | | 1 medium |  | |  |  |  |
| **508** | Fruit salad | | 1 cup |  | |  |  |  |
| **509** | Dried fruits-Dates/ Resins | | 1 handful |  | |  |  |  |
| **600** | **Beverages** | | | | | | | |
| **601** | Nestamalt /Viva/Milo | | 1 glass |  | |  |  |  |
| **602** | Full cream milk | | 1 glass |  | |  |  |  |
| **603** | Non fat milk | | 1 glass |  | |  |  |  |
| **604** | Fresh milk | | 1 glass |  | |  |  |  |
| **605** | Plain tea | | 1 cup |  | |  |  |  |
| **606** | Coffee | | 1 cup |  | |  |  |  |
| **607** | Fizzy drinks | | 200 ml |  | |  |  |  |
| **608** | Cordial/Smack/Colman | | 1 glass |  | |  |  |  |
| **609** | Kola kada/rice kanji | | 1 glass |  | |  |  |  |
| **700** | **Miscellaneous** | | | | | | | |
| **701** | Yoghurt | | 1 cup |  | |  |  |  |
| **702** | Ice cream | | 1 cup |  | |  |  |  |
| **703** | Curd | | 1 cup |  | |  |  |  |
| **704** | Cheese | | 1 wedge |  | |  |  |  |
| **705** | Un-sweeten Biscuits | | 1 |  | |  |  |  |
| **706** | Cream/Sweet biscuits | | 1 |  | |  |  |  |
| **707** | Cake | | 1 medium size |  | |  |  |  |
| **Code**  No | | **Food Type** |  |  |  |  |  |  | | --- | --- | --- | --- | --- | --- | --- | | | **Portion size** | **No of units taken at a time** | | **Per**  **Day** | **Per**  **Week** | **Per**  **Month** |
| **708** | Watalappam/Pudding | | 1 cup |  | |  |  |  |
| **709** | Sweets | | 1 medium |  | |  |  |  |
| **710** | Sugar | | 1 tea spoon |  | |  |  |  |
| **711** | Toffee | | 1 |  | |  |  |  |
| **712** | Chocolate | | 1 |  | |  |  |  |
| **713** | Butter/Margarine | | 1 tea spoon |  | |  |  |  |
| **714** | Jam | | 1 tea spoon |  | |  |  |  |
| **715** | Tomato sauce | | 1 table spoon |  | |  |  |  |
| **716** | Mixtures/salted nuts | | 1 table spoon |  | |  |  |  |
| **800** | **Alcohol** | | | | | | | |
| **801** | Spirit | | 25 ml |  | |  |  |  |
| **802** | Beer | | 1 pint |  | |  |  |  |

1. **VISUAL ANALOGUE SCALES (VAS)**

- How **HUNGRY** do you feel *at this moment*?

**Not at all Very**

**HUNGRY HUNGRY**

- How **FULL** do you feel *at this moment*?

**Not at all Very**

**FULL FULL**

- How **STRONG** is your desire to eat *at this moment*?

**Not at all Very**

**STRONG STRONG**

- How **MUCH FOOD** do you think you could eat *at this moment*?

**None A large**

**Amount**

**Investigator’s Signature:___________________________ Date:________________**

1. **COMPLIANCE**

**Total number of medicine/tablets expected to be used in last 30days :________**

**Total number of medicine/tablets actually used last 30 days :________**

**Total number of medicine /tablets missed/unused in last 30 days :________**

1. **ANALYSIS OF ADVERSE EVENT**

**Adverse events: Yes No**

**If yes, please specify**

| **Sr no** | **Adverse Effect (AE)** | **Severity*** | **Causality @** | **Drug responsible for AE** |
| --- | --- | --- | --- | --- |
|  |  |  |  |  |
|  |  |  |  |  |
|  |  |  |  |  |
|  |  |  |  |  |
|  |  |  |  |  |
| *** - Mild, Moderate, Severe**  **@ - Related (R), Possibly Related(PR), Definitely Related(DR) and Unlikely to be related (UR)** | | | | |

**Any serious adverse event (SAE): Yes No**

***If yes, please fill the SAE form enclosed at the end of the case report form and send to sponsor immediately.***

**Details of SAE:**

**_________________________________________________________________________________________________________________________________________________________________________________________________________________________________**

**Did the patient discontinue the study for any reason? Yes No**

**If yes, give reason:___________________________________________________________**

**Any missed doses: Yes No**

**Whether modification of test/reference drug is required? Yes No**

**Investigator’s Signature :___________________________ Date:________________**

**VISIT 2 – SECOND VISIT**

**(After 3 months therapy)**

**OPD No.______________ Patient Initials: _________________ Date:___/___/___**

1. **PRESENT COMPLAINTS**

______________________________________________________________________________________________________________________________________________________________________________________________________________

1. **GENERAL PHYSICAL EXAMINATION**

**Temperature =__________ ^0^C**

**Anemia Jaundice Liver___________ Spleen ___________**

**Any other abnormality _________________________________________________**

1. **SYSTEMIC EXAMIANTION**

**Respiratory system:____________________________________________________**

**__________________________________________________________________________________________________________________________________________**

**Gastrointestinal system:________________________________________________**

**__________________________________________________________________________________________________________________________________________**

**Central Nervous system:________________________________________________**

**__________________________________________________________________________________________________________________________________________**

1. **CARDIOVASCULAR SYSTEM EXAMINATION:**

**a) Pulse Examination:**

**Rate :___________________________________________/ minute**

**Rhythm :__________________________________________________**

**Volume :__________________________________________________**

**Force :__________________________________________________**

**b) Blood Pressure (Sitting position)**

**Subject has to take 30 minutes rest before recording Blood Pressure.**

**Systolic blood pressure (SBP) :____________________ mm of Hg**

**Diastolic blood pressure (DBP) :____________________ mm of Hg**

**Mean blood pressure (MBP) :____________________ mm of Hg**

Mean blood pressure = DBP + 1/3 (SBP – DBP)

**c)Auscultation:_____________________________________________________________________________________________________________________________**

1. **ANTHROPOMETRIC MEASUREMENT**

| **SR. NO** | **PARAMETER** | **MEASUREMENT** |
| --- | --- | --- |
| 1. | Height (cm) |  |
| 2. | Body weight (kg) |  |
| 3. | Waist circumference (cm) |  |
| 4. | Hip circumference (cm) |  |

1. **LABORATORY INVESTIGATIONS**

| **SR No** | **PARAMETER** | **OBSERVED VALUE** | **NORMAL RANGE** |
| --- | --- | --- | --- |
| 1. | Hemoglobin |  |  |
| 2. | RBC Count |  |  |
| 3. | WBC Count |  |  |
| 4. | Differential WBC Count | N______ E______ B_____ L_____ M_____ |  |
| 5. | Platelet Count |  |  |
| 6. | LDL |  |  |
| 7. | HDL |  |  |
| 8. | Total Cholesterol |  |  |
| 9. | Triglycerides |  |  |
| 10. | Fasting Blood Glucose level |  |  |
| 11. | OGTT |  |  |
| 12. | HbA1c |  |  |
| 13. | Serum Insulin |  |  |
| 14. | Serum Zinc |  |  |

1. **FOOD FREQUENCY QUESTIONNAIRE**

1.When you eat **RICE**, did you usually eat:

| **Figure A**  **A** | **Figure B** | **Figure C** |
| --- | --- | --- |

| Less than A | A | Between A & B | B | Between A & B | C | More than C |
| --- | --- | --- | --- | --- | --- | --- |

2. When you eat **VEGETABLE**, did you usually eat:

| **Figure A**  **B** | | | **Figure B** | | | **Figure C** | | |  |
| --- | --- | --- | --- | --- | --- | --- | --- | --- | --- |
| Less than A | A | Between A & B | | B | Between A & B | | C | More than C | |

3. When you eat **MEAT**, did you usually eat:

| **Figure A**  **C** | | | **Figure B** | | | **Figure C** | | |  |
| --- | --- | --- | --- | --- | --- | --- | --- | --- | --- |
| Less than A | A | Between A & B | | B | Between A & B | | C | More than C | |

4. When you eat **DHAL**, did you usually eat:

| **Figure A**  **D** | | | **Figure B** | | | **Figure C** | | |  |
| --- | --- | --- | --- | --- | --- | --- | --- | --- | --- |
| Less than A | A | Between A & B | | B | Between A & B | | C | More than C | |

| **Code**  **No** | **Food Type** | | **Portion size** | **No of units taken at a time** | | **Per**  **Day** | **Per**  **Week** | **Per**  **Month** |
| --- | --- | --- | --- | --- | --- | --- | --- | --- |
| **100** | **Cereals or equivalents** | | | | | | | |
| **101** | Unpolished Rice (Red Kakulu) | According to picture **A** | | |  |  |  |  |
| **102** | Polished rice | According to picture **A** | | |  |  |  |  |
| **103** | Bread | 1 slice | | |  |  |  |  |
| **104** | String hoppers | 1 medium size | | |  |  |  |  |
| **105** | Roti | 1 medium | | |  |  |  |  |
| **106** | Pittu | 5 cm length piece | | |  |  |  |  |
| **107** | Noodles/Macaroni/Pasta | According to picture **A** | | |  |  |  |  |
| **108** | Hoppers | 1 medium | | |  |  |  |  |
| **109** | Milk rice | 1 piece | | |  |  |  |  |
| **110** | Bun & Bakery products | 1 medium | | |  |  |  |  |
| **111** | Thosai | 1 medium | | |  |  |  |  |
| **112** | Potato | 1 table spoon | | |  |  |  |  |
| **113** | Manioc | 1 medium coconut spoon | | |  |  |  |  |
| **114** | Boiled sweet potato | 1 medium coconut spoon | | |  |  |  |  |
| **115** | Jak | 1 medium coconut spoon | | |  |  |  |  |
| **116** | Breadfruit | 1 medium coconut spoon | | |  |  |  |  |
| **117** | Ash plantain | 1 table spoon | | |  |  |  |  |
| **200** | **Vegetables** | | | | | | | |
| **201** | Coconut sambol | | According to picture **B** | |  |  |  |  |
| **202** | Beans/ long beans/ wing beans curry | |  | |  |  |  |  |
| **203** | Cabbage curry | |  | |  |  |  |  |
| **204** | Pumpkin curry | |  | |  |  |  |  |
| **205** | Brinjol curry | |  | |  |  |  |  |
| **206** | Polos curry/mallum | |  | |  |  |  |  |
| **207** | Carrot curry | |  | |  |  |  |  |
| **208** | Kohila curry | |  | |  |  |  |  |
| **209** | Kehelmuwa curry | |  | |  |  |  |  |
| **Code**  No | | **Food Type** |  |  |  |  |  |  | | --- | --- | --- | --- | --- | --- | --- | | | **Portion size** | | **No of units taken at a time** | **Per**  **Day** | **Per**  **Week** | **Per**  **Month** |
| **210** | Leaks curry | |  | |  |  |  |  |
| **211** | Bittergourd curry | |  | |  |  |  |  |
| **212** | Radish/Nohkol | |  | |  |  |  |  |
| **213** | Snakegourd/Ridgegourd | |  | |  |  |  |  |
| **214** | Beetroot curry | |  | |  |  |  |  |
| **215** | Mushroom curry | |  | |  |  |  |  |
| **216** | Tomato curry | |  | |  |  |  |  |
| **217** | Cucumber/Kakiri curry | |  | |  |  |  |  |
| **218** | Ladies fingers curry | |  | |  |  |  |  |
| **219** | Common mallum | |  | |  |  |  |  |
| **220** | Vegetable soup | | 1 cup | |  |  |  |  |
| **300** | **Pulses** | | | | | | | |
| **301** | Dhal curry (thick) | | According to picture **D** |  | |  |  |  |
| **302** | Dhal curry (watery) | | According to picture **D** |  | |  |  |  |
| **303** | Soya curry | |  |  | |  |  |  |
| **304** | Chick peas | |  |  | |  |  |  |
| **305** | Green grams | |  |  | |  |  |  |
| **306** | Samaposha | |  |  | |  |  |  |
| **400** | **Meat/Poultry and meat/Poultry products** | | | | | | | |
| **401** | Chicken | | According to picture **C** |  | |  |  |  |
| **402** | Beef/Pork/Mutton | | According to picture **C** |  | |  |  |  |
| **403** | Meat balls/sausages | | 1 medium size |  | |  |  |  |
| **404** | Egg boiled/Omelet | | 1 |  | |  |  |  |
| **405** | Dry fish /Sprat | | 1 table spoon |  | |  |  |  |
| **406** | Fish | | 1 medium piece |  | |  |  |  |
| **407** | Salmon | | 1 medium piece |  | |  |  |  |
| **408** | prawn/cuttlefish/crabs | | 1 medium |  | |  |  |  |
| **Code**  No | | **Food Type** |  |  |  |  |  |  | | --- | --- | --- | --- | --- | --- | --- | | | **Portion size** | **No of units taken at a time** | | **Per**  **Day** | **Per**  **Week** | **Per**  **Month** |
| **500** | **Fruits** | | | | | | | |
| **501** | Banana | | 1 medium |  | |  |  |  |
| **502** | Papaya | | 1 piece |  | |  |  |  |
| **503** | Apple | | 1 medium |  | |  |  |  |
| **504** | Mango | | 1 medium |  | |  |  |  |
| **505** | Orange | | 1 medium |  | |  |  |  |
| **506** | Pineapple | | Medium size |  | |  |  |  |
| **507** | Guava | | 1 medium |  | |  |  |  |
| **508** | Fruit salad | | 1 cup |  | |  |  |  |
| **509** | Dried fruits-Dates/ Resins | | 1 handful |  | |  |  |  |
| **600** | **Beverages** | | | | | | | |
| **601** | Nestamalt /Viva/Milo | | 1 glass |  | |  |  |  |
| **602** | Full cream milk | | 1 glass |  | |  |  |  |
| **603** | Non fat milk | | 1 glass |  | |  |  |  |
| **604** | Fresh milk | | 1 glass |  | |  |  |  |
| **605** | Plain tea | | 1 cup |  | |  |  |  |
| **606** | Coffee | | 1 cup |  | |  |  |  |
| **607** | Fizzy drinks | | 200 ml |  | |  |  |  |
| **608** | Cordial/Smack/Colman | | 1 glass |  | |  |  |  |
| **609** | Kola kada/rice kanji | | 1 glass |  | |  |  |  |
| **700** | **Miscellaneous** | | | | | | | |
| **701** | Yoghurt | | 1 cup |  | |  |  |  |
| **702** | Ice cream | | 1 cup |  | |  |  |  |
| **703** | Curd | | 1 cup |  | |  |  |  |
| **704** | Cheese | | 1 wedge |  | |  |  |  |
| **705** | Un-sweeten Biscuits | | 1 |  | |  |  |  |
| **706** | Cream/Sweet biscuits | | 1 |  | |  |  |  |
| **707** | Cake | | 1 medium size |  | |  |  |  |
| **Code**  No | | **Food Type** |  |  |  |  |  |  | | --- | --- | --- | --- | --- | --- | --- | | | **Portion size** | **No of units taken at a time** | | **Per**  **Day** | **Per**  **Week** | **Per**  **Month** |
| **708** | Watalappam/Pudding | | 1 cup |  | |  |  |  |
| **709** | Sweets | | 1 medium |  | |  |  |  |
| **710** | Sugar | | 1 tea spoon |  | |  |  |  |
| **711** | Toffee | | 1 |  | |  |  |  |
| **712** | Chocolate | | 1 |  | |  |  |  |
| **713** | Butter/Margarine | | 1 tea spoon |  | |  |  |  |
| **714** | Jam | | 1 tea spoon |  | |  |  |  |
| **715** | Tomato sauce | | 1 table spoon |  | |  |  |  |
| **716** | Mixtures/salted nuts | | 1 table spoon |  | |  |  |  |
| **800** | **Alcohol** | | | | | | | |
| **801** | Spirit | | 25 ml |  | |  |  |  |
| **802** | Beer | | 1 pint |  | |  |  |  |

1. **VISUAL ANALOGUE SCALES (VAS)**

- How **HUNGRY** do you feel *at this moment*?

**Not at all Very**

**HUNGRY HUNGRY**

- How **FULL** do you feel *at this moment*?

**Not at all Very**

**FULL FULL**

- How **STRONG** is your desire to eat *at this moment*?

**Not at all Very**

**STRONG STRONG**

- How **MUCH FOOD** do you think you could eat *at this moment*?

**None A large Amount**

**Investigator’s Signature:__________________________ Date:________________**

1. **COMPLIANCE**

**Total number of medicine/tablets expected to be used in last 30days :________**

**Total number of medicine/tablets actually used last 30 days :________**

**Total number of medicine /tablets missed/unused in last 30 days :________**

1. **ANALYSIS OF ADVERSE EVENT**

**Adverse events: Yes No**

**If yes, please specify**

| **Sr no** | **Adverse Effect (AE)** | **Severity*** | **Causality @** | **Drug responsible for AE** |
| --- | --- | --- | --- | --- |
|  |  |  |  |  |
|  |  |  |  |  |
|  |  |  |  |  |
|  |  |  |  |  |
|  |  |  |  |  |
| *** - Mild, Moderate, Severe**  **@ - Related (R), Possibly Related(PR), Definitely Related(DR) and Unlikely to be related (UR)** | | | | |

**Any serious adverse event (SAE): Yes No**

***If yes, please fill the SAE form enclosed at the end of the case report form and send to sponsor immediately.***

**Details of SAE:**

**_________________________________________________________________________________________________________________________________________________________________________________________________________________________________**

**Did the patient discontinue the study for any reason? Yes No**

**If yes, give reason:___________________________________________________________**

**Any missed doses: Yes No**

**Whether modification of test/reference drug is required? Yes No**

**Investigator’s Signature :___________________________ Date:________________**

**VISIT 3 – THIRD VISIT**

**(After 6 months therapy)**

**OPD No.______________ Patient Initials: _________________ Date:___/___/___**

1. **PRESENT COMPLAINTS**

______________________________________________________________________________________________________________________________________________________________________________________________________________

1. **GENERAL PHYSICAL EXAMINATION**

**Temperature =__________ ^0^C**

**Anemia Jaundice Liver___________ Spleen ___________**

**Any other abnormality _________________________________________________**

1. **SYSTEMIC EXAMIANTION**

**Respiratory system:____________________________________________________**

**__________________________________________________________________________________________________________________________________________**

**Gastrointestinal system:________________________________________________**

**__________________________________________________________________________________________________________________________________________**

**Central Nervous system:________________________________________________**

**__________________________________________________________________________________________________________________________________________**

1. **CARDIOVASCULAR SYSTEM EXAMINATION:**

**a) Pulse Examination:**

**Rate :___________________________________________/ minute**

**Rhythm :__________________________________________________**

**Volume :__________________________________________________**

**Force :__________________________________________________**

**b) Blood Pressure (Sitting position)**

**Subject has to take 30 minutes rest before recording Blood Pressure.**

**Systolic blood pressure (SBP) :____________________ mm of Hg**

**Diastolic blood pressure (DBP) :____________________ mm of Hg**

**Mean blood pressure (MBP) :____________________ mm of Hg**

Mean blood pressure = DBP + 1/3 (SBP – DBP)

**c)Auscultation:_____________________________________________________________________________________________________________________________**

1. **ANTHROPOMETRIC MEASUREMENT**

| **SR. NO** | **PARAMETER** | **MEASUREMENT** |
| --- | --- | --- |
| 1. | Height (cm) |  |
| 2. | Body weight (kg) |  |
| 3. | Waist circumference (cm) |  |
| 4. | Hip circumference (cm) |  |

1. **LABORATORY INVESTIGATIONS**

| **SR No** | **PARAMETER** | **OBSERVED VALUE** | **NORMAL RANGE** |
| --- | --- | --- | --- |
| 1. | Hemoglobin |  |  |
| 2. | RBC Count |  |  |
| 3. | WBC Count |  |  |
| 4. | Differential WBC Count | N______ E______ B_____ L_____ M_____ |  |
| 5. | Platelet Count |  |  |
| 6. | LDL |  |  |
| 7. | HDL |  |  |
| 8. | Total Cholesterol |  |  |
| 9. | Triglycerides |  |  |
| 10. | Fasting Blood Glucose level |  |  |
| 11. | OGTT |  |  |
| 12. | HbA1c |  |  |
| 13. | Serum Insulin |  |  |
| 14. | Serum Zinc |  |  |
| 15. | ALT |  |  |
| 16. | AST |  |  |
| 17. | Total bilirubin |  |  |
| 18. | Creatinine |  |  |

1. **FOOD FREQUENCY QUESTIONNAIRE**

1.When you eat **RICE**, did you usually eat:

| **Figure A**  **A** | **Figure B** | **Figure C** |
| --- | --- | --- |

| Less than A | A | Between A & B | B | Between A & B | C | More than C |
| --- | --- | --- | --- | --- | --- | --- |

2. When you eat **VEGETABLE**, did you usually eat:

| **Figure A**  **B** | | | **Figure B** | | | **Figure C** | | |  |
| --- | --- | --- | --- | --- | --- | --- | --- | --- | --- |
| Less than A | A | Between A & B | | B | Between A & B | | C | More than C | |

3. When you eat **MEAT**, did you usually eat:

| **Figure A**  **C** | | | **Figure B** | | | **Figure C** | | |  |
| --- | --- | --- | --- | --- | --- | --- | --- | --- | --- |
| Less than A | A | Between A & B | | B | Between A & B | | C | More than C | |

4. When you eat **DHAL**, did you usually eat:

| **Figure A**  **D** | | | **Figure B** | | | **Figure C** | | |  |
| --- | --- | --- | --- | --- | --- | --- | --- | --- | --- |
| Less than A | A | Between A & B | | B | Between A & B | | C | More than C | |

| **Code**  **No** | **Food Type** | | **Portion size** | **No of units taken at a time** | | **Per**  **Day** | **Per**  **Week** | **Per**  **Month** |
| --- | --- | --- | --- | --- | --- | --- | --- | --- |
| **100** | **Cereals or equivalents** | | | | | | | |
| **101** | Unpolished Rice (Red Kakulu) | According to picture **A** | | |  |  |  |  |
| **102** | Polished rice | According to picture **A** | | |  |  |  |  |
| **103** | Bread | 1 slice | | |  |  |  |  |
| **104** | String hoppers | 1 medium size | | |  |  |  |  |
| **105** | Roti | 1 medium | | |  |  |  |  |
| **106** | Pittu | 5 cm length piece | | |  |  |  |  |
| **107** | Noodles/Macaroni/Pasta | According to picture **A** | | |  |  |  |  |
| **108** | Hoppers | 1 medium | | |  |  |  |  |
| **109** | Milk rice | 1 piece | | |  |  |  |  |
| **110** | Bun & Bakery products | 1 medium | | |  |  |  |  |
| **111** | Thosai | 1 medium | | |  |  |  |  |
| **112** | Potato | 1 table spoon | | |  |  |  |  |
| **113** | Manioc | 1 medium coconut spoon | | |  |  |  |  |
| **114** | Boiled sweet potato | 1 medium coconut spoon | | |  |  |  |  |
| **115** | Jak | 1 medium coconut spoon | | |  |  |  |  |
| **116** | Breadfruit | 1 medium coconut spoon | | |  |  |  |  |
| **117** | Ash plantain | 1 table spoon | | |  |  |  |  |
| **200** | **Vegetables** | | | | | | | |
| **201** | Coconut sambol | | According to picture **B** | |  |  |  |  |
| **202** | Beans/ long beans/ wing beans curry | |  | |  |  |  |  |
| **203** | Cabbage curry | |  | |  |  |  |  |
| **204** | Pumpkin curry | |  | |  |  |  |  |
| **205** | Brinjol curry | |  | |  |  |  |  |
| **206** | Polos curry/mallum | |  | |  |  |  |  |
| **207** | Carrot curry | |  | |  |  |  |  |
| **208** | Kohila curry | |  | |  |  |  |  |
| **209** | Kehelmuwa curry | |  | |  |  |  |  |
| **Code**  No | | **Food Type** |  |  |  |  |  |  | | --- | --- | --- | --- | --- | --- | --- | | | **Portion size** | | **No of units taken at a time** | **Per**  **Day** | **Per**  **Week** | **Per**  **Month** |
| **210** | Leaks curry | |  | |  |  |  |  |
| **211** | Bittergourd curry | |  | |  |  |  |  |
| **212** | Radish/Nohkol | |  | |  |  |  |  |
| **213** | Snakegourd/Ridgegourd | |  | |  |  |  |  |
| **214** | Beetroot curry | |  | |  |  |  |  |
| **215** | Mushroom curry | |  | |  |  |  |  |
| **216** | Tomato curry | |  | |  |  |  |  |
| **217** | Cucumber/Kakiri curry | |  | |  |  |  |  |
| **218** | Ladies fingers curry | |  | |  |  |  |  |
| **219** | Common mallum | |  | |  |  |  |  |
| **220** | Vegetable soup | | 1 cup | |  |  |  |  |
| **300** | **Pulses** | | | | | | | |
| **301** | Dhal curry (thick) | | According to picture **D** |  | |  |  |  |
| **302** | Dhal curry (watery) | | According to picture **D** |  | |  |  |  |
| **303** | Soya curry | |  |  | |  |  |  |
| **304** | Chick peas | |  |  | |  |  |  |
| **305** | Green grams | |  |  | |  |  |  |
| **306** | Samaposha | |  |  | |  |  |  |
| **400** | **Meat/Poultry and meat/Poultry products** | | | | | | | |
| **401** | Chicken | | According to picture **C** |  | |  |  |  |
| **402** | Beef/Pork/Mutton | | According to picture **C** |  | |  |  |  |
| **403** | Meat balls/sausages | | 1 medium size |  | |  |  |  |
| **404** | Egg boiled/Omelet | | 1 |  | |  |  |  |
| **405** | Dry fish /Sprat | | 1 table spoon |  | |  |  |  |
| **406** | Fish | | 1 medium piece |  | |  |  |  |
| **407** | Salmon | | 1 medium piece |  | |  |  |  |
| **408** | prawn/cuttlefish/crabs | | 1 medium |  | |  |  |  |
| **Code**  No | | **Food Type** |  |  |  |  |  |  | | --- | --- | --- | --- | --- | --- | --- | | | **Portion size** | **No of units taken at a time** | | **Per**  **Day** | **Per**  **Week** | **Per**  **Month** |
| **500** | **Fruits** | | | | | | | |
| **501** | Banana | | 1 medium |  | |  |  |  |
| **502** | Papaya | | 1 piece |  | |  |  |  |
| **503** | Apple | | 1 medium |  | |  |  |  |
| **504** | Mango | | 1 medium |  | |  |  |  |
| **505** | Orange | | 1 medium |  | |  |  |  |
| **506** | Pineapple | | Medium size |  | |  |  |  |
| **507** | Guava | | 1 medium |  | |  |  |  |
| **508** | Fruit salad | | 1 cup |  | |  |  |  |
| **509** | Dried fruits-Dates/ Resins | | 1 handful |  | |  |  |  |
| **600** | **Beverages** | | | | | | | |
| **601** | Nestamalt /Viva/Milo | | 1 glass |  | |  |  |  |
| **602** | Full cream milk | | 1 glass |  | |  |  |  |
| **603** | Non fat milk | | 1 glass |  | |  |  |  |
| **604** | Fresh milk | | 1 glass |  | |  |  |  |
| **605** | Plain tea | | 1 cup |  | |  |  |  |
| **606** | Coffee | | 1 cup |  | |  |  |  |
| **607** | Fizzy drinks | | 200 ml |  | |  |  |  |
| **608** | Cordial/Smack/Colman | | 1 glass |  | |  |  |  |
| **609** | Kola kada/rice kanji | | 1 glass |  | |  |  |  |
| **700** | **Miscellaneous** | | | | | | | |
| **701** | Yoghurt | | 1 cup |  | |  |  |  |
| **702** | Ice cream | | 1 cup |  | |  |  |  |
| **703** | Curd | | 1 cup |  | |  |  |  |
| **704** | Cheese | | 1 wedge |  | |  |  |  |
| **705** | Un-sweeten Biscuits | | 1 |  | |  |  |  |
| **706** | Cream/Sweet biscuits | | 1 |  | |  |  |  |
| **707** | Cake | | 1 medium size |  | |  |  |  |
| **Code**  No | | **Food Type** |  |  |  |  |  |  | | --- | --- | --- | --- | --- | --- | --- | | | **Portion size** | **No of units taken at a time** | | **Per**  **Day** | **Per**  **Week** | **Per**  **Month** |
| **708** | Watalappam/Pudding | | 1 cup |  | |  |  |  |
| **709** | Sweets | | 1 medium |  | |  |  |  |
| **710** | Sugar | | 1 tea spoon |  | |  |  |  |
| **711** | Toffee | | 1 |  | |  |  |  |
| **712** | Chocolate | | 1 |  | |  |  |  |
| **713** | Butter/Margarine | | 1 tea spoon |  | |  |  |  |
| **714** | Jam | | 1 tea spoon |  | |  |  |  |
| **715** | Tomato sauce | | 1 table spoon |  | |  |  |  |
| **716** | Mixtures/salted nuts | | 1 table spoon |  | |  |  |  |
| **800** | **Alcohol** | | | | | | | |
| **801** | Spirit | | 25 ml |  | |  |  |  |
| **802** | Beer | | 1 pint |  | |  |  |  |

1. **VISUAL ANALOGUE SCALES (VAS)**

- How **HUNGRY** do you feel *at this moment*?

**Not at all Very**

**HUNGRY HUNGRY**

- How **FULL** do you feel *at this moment*?

**Not at all Very**

**FULL FULL**

- How **STRONG** is your desire to eat *at this moment*?

**Not at all Very**

**STRONG STRONG**

- How **MUCH FOOD** do you think you could eat *at this moment*?

**None A large Amount**

**Investigator’s Signature:__________________________ Date:________________**

1. **COMPLIANCE**

**Total number of medicine/tablets expected to be used in last 30days :________**

**Total number of medicine/tablets actually used last 30 days :________**

**Total number of medicine /tablets missed/unused in last 30 days :________**

1. **ANALYSIS OF ADVERSE EVENT**

**Adverse events: Yes No**

**If yes, please specify**

| **Sr no** | **Adverse Effect (AE)** | **Severity*** | **Causality @** | **Drug responsible for AE** |
| --- | --- | --- | --- | --- |
|  |  |  |  |  |
|  |  |  |  |  |
|  |  |  |  |  |
|  |  |  |  |  |
|  |  |  |  |  |
| *** - Mild, Moderate, Severe**  **@ - Related (R), Possibly Related(PR), Definitely Related(DR) and Unlikely to be related (UR)** | | | | |

**Any serious adverse event (SAE): Yes No**

***If yes, please fill the SAE form enclosed at the end of the case report form and send to sponsor immediately.***

**Details of SAE:**

**_________________________________________________________________________________________________________________________________________________________________________________________________________________________________**

**Did the patient discontinue the study for any reason? Yes No**

**If yes, give reason:___________________________________________________________**

**Any missed doses: Yes No**

**Whether modification of test/reference drug is required? Yes No**

**Investigator’s Signature :___________________________ Date:________________**

**VISIT 4 – FINAL VISIT**

**(After 12 months therapy)**

**OPD No.______________ Patient Initials: _________________ Date:___/___/___**

1. **PRESENT COMPLAINTS**

______________________________________________________________________________________________________________________________________________________________________________________________________________

1. **GENERAL PHYSICAL EXAMINATION**

**Temperature =__________ ^0^C**

**Anemia Jaundice Liver___________ Spleen ___________**

**Any other abnormality _________________________________________________**

1. **SYSTEMIC EXAMIANTION**

**Respiratory system:____________________________________________________**

**__________________________________________________________________________________________________________________________________________**

**Gastrointestinal system:________________________________________________**

**__________________________________________________________________________________________________________________________________________**

**Central Nervous system:________________________________________________**

**__________________________________________________________________________________________________________________________________________**

1. **CARDIOVASCULAR SYSTEM EXAMINATION:**

**a) Pulse Examination:**

**Rate :___________________________________________/ minute**

**Rhythm :__________________________________________________**

**Volume :__________________________________________________**

**Force :__________________________________________________**

**b) Blood Pressure (Sitting position)**

**Subject has to take 30 minutes rest before recording Blood Pressure.**

**Systolic blood pressure (SBP) :____________________ mm of Hg**

**Diastolic blood pressure (DBP) :____________________ mm of Hg**

**Mean blood pressure (MBP) :____________________ mm of Hg**

Mean blood pressure = DBP + 1/3 (SBP – DBP)

**c)Auscultation:_____________________________________________________________________________________________________________________________**

1. **ANTHROPOMETRIC MEASUREMENT**

| **SR. NO** | **PARAMETER** | **MEASUREMENT** |
| --- | --- | --- |
| 1. | Height (cm) |  |
| 2. | Body weight (kg) |  |
| 3. | Waist circumference (cm) |  |
| 4. | Hip circumference (cm) |  |

1. **ELECTROCARDIOGRAM FINDINGS**:

Heart Rate________________________________________________________

Rhythm/ Ectopics __________________________________________________

PR interval________________________________________________________

ST segment_______________________________________________________

QRS complex______________________________________________________

Other_____________________________________________________________

1. LABORATORY INVESTIGATIONS

| **SR No** | **PARAMETER** | **OBSERVED VALUE** | **NORMAL RANGE** |
| --- | --- | --- | --- |
| 1. | Hemoglobin |  |  |
| 2. | RBC Count |  |  |
| 3. | WBC Count |  |  |
| 4. | Differential WBC Count | N______ E______ B_____ L_____ M_____ |  |
| 5. | Platelet Count |  |  |
| 6. | LDL |  |  |
| 7. | HDL |  |  |
| 8. | Total Cholesterol |  |  |
| 9. | Triglycerides |  |  |
| 10. | Fasting Blood Glucose level |  |  |
| 11. | OGTT |  |  |
| 12. | HbA1c |  |  |
| 13. | Serum Insulin |  |  |
| 14. | Serum Zinc |  |  |
| 15. | ALT |  |  |
| 16. | AST |  |  |
| 17. | Total bilirubin |  |  |
| 18. | Creatinine |  |  |

1. **FOOD FREQUENCY QUESTIONNAIRE**

1.When you eat **RICE**, did you usually eat:

| **Figure A**  **A** | **Figure B** | **Figure C** |
| --- | --- | --- |

| Less than A | A | Between A & B | B | Between A & B | C | More than C |
| --- | --- | --- | --- | --- | --- | --- |

2. When you eat **VEGETABLE**, did you usually eat:

| **Figure A**  **B** | | | **Figure B** | | | **Figure C** | | |  |
| --- | --- | --- | --- | --- | --- | --- | --- | --- | --- |
| Less than A | A | Between A & B | | B | Between A & B | | C | More than C | |

3. When you eat **MEAT**, did you usually eat:

| **Figure A**  **C** | | | **Figure B** | | | **Figure C** | | |  |
| --- | --- | --- | --- | --- | --- | --- | --- | --- | --- |
| Less than A | A | Between A & B | | B | Between A & B | | C | More than C | |

4. When you eat **DHAL**, did you usually eat:

| **Figure A**  **D** | | | **Figure B** | | | **Figure C** | | |  |
| --- | --- | --- | --- | --- | --- | --- | --- | --- | --- |
| Less than A | A | Between A & B | | B | Between A & B | | C | More than C | |

| **Code**  **No** | **Food Type** | | **Portion size** | **No of units taken at a time** | | **Per**  **Day** | **Per**  **Week** | **Per**  **Month** |
| --- | --- | --- | --- | --- | --- | --- | --- | --- |
| **100** | **Cereals or equivalents** | | | | | | | |
| **101** | Unpolished Rice (Red Kakulu) | According to picture **A** | | |  |  |  |  |
| **102** | Polished rice | According to picture **A** | | |  |  |  |  |
| **103** | Bread | 1 slice | | |  |  |  |  |
| **104** | String hoppers | 1 medium size | | |  |  |  |  |
| **105** | Roti | 1 medium | | |  |  |  |  |
| **106** | Pittu | 5 cm length piece | | |  |  |  |  |
| **107** | Noodles/Macaroni/Pasta | According to picture **A** | | |  |  |  |  |
| **108** | Hoppers | 1 medium | | |  |  |  |  |
| **109** | Milk rice | 1 piece | | |  |  |  |  |
| **110** | Bun & Bakery products | 1 medium | | |  |  |  |  |
| **111** | Thosai | 1 medium | | |  |  |  |  |
| **112** | Potato | 1 table spoon | | |  |  |  |  |
| **113** | Manioc | 1 medium coconut spoon | | |  |  |  |  |
| **114** | Boiled sweet potato | 1 medium coconut spoon | | |  |  |  |  |
| **115** | Jak | 1 medium coconut spoon | | |  |  |  |  |
| **116** | Breadfruit | 1 medium coconut spoon | | |  |  |  |  |
| **117** | Ash plantain | 1 table spoon | | |  |  |  |  |
| **200** | **Vegetables** | | | | | | | |
| **201** | Coconut sambol | | According to picture **B** | |  |  |  |  |
| **202** | Beans/ long beans/ wing beans curry | |  | |  |  |  |  |
| **203** | Cabbage curry | |  | |  |  |  |  |
| **204** | Pumpkin curry | |  | |  |  |  |  |
| **205** | Brinjol curry | |  | |  |  |  |  |
| **206** | Polos curry/mallum | |  | |  |  |  |  |
| **207** | Carrot curry | |  | |  |  |  |  |
| **208** | Kohila curry | |  | |  |  |  |  |
| **209** | Kehelmuwa curry | |  | |  |  |  |  |
| **Code**  No | | **Food Type** |  |  |  |  |  |  | | --- | --- | --- | --- | --- | --- | --- | | | **Portion size** | | **No of units taken at a time** | **Per**  **Day** | **Per**  **Week** | **Per**  **Month** |
| **210** | Leaks curry | |  | |  |  |  |  |
| **211** | Bittergourd curry | |  | |  |  |  |  |
| **212** | Radish/Nohkol | |  | |  |  |  |  |
| **213** | Snakegourd/Ridgegourd | |  | |  |  |  |  |
| **214** | Beetroot curry | |  | |  |  |  |  |
| **215** | Mushroom curry | |  | |  |  |  |  |
| **216** | Tomato curry | |  | |  |  |  |  |
| **217** | Cucumber/Kakiri curry | |  | |  |  |  |  |
| **218** | Ladies fingers curry | |  | |  |  |  |  |
| **219** | Common mallum | |  | |  |  |  |  |
| **220** | Vegetable soup | | 1 cup | |  |  |  |  |
| **300** | **Pulses** | | | | | | | |
| **301** | Dhal curry (thick) | | According to picture **D** |  | |  |  |  |
| **302** | Dhal curry (watery) | | According to picture **D** |  | |  |  |  |
| **303** | Soya curry | |  |  | |  |  |  |
| **304** | Chick peas | |  |  | |  |  |  |
| **305** | Green grams | |  |  | |  |  |  |
| **306** | Samaposha | |  |  | |  |  |  |
| **400** | **Meat/Poultry and meat/Poultry products** | | | | | | | |
| **401** | Chicken | | According to picture **C** |  | |  |  |  |
| **402** | Beef/Pork/Mutton | | According to picture **C** |  | |  |  |  |
| **403** | Meat balls/sausages | | 1 medium size |  | |  |  |  |
| **404** | Egg boiled/Omelet | | 1 |  | |  |  |  |
| **405** | Dry fish /Sprat | | 1 table spoon |  | |  |  |  |
| **406** | Fish | | 1 medium piece |  | |  |  |  |
| **407** | Salmon | | 1 medium piece |  | |  |  |  |
| **408** | prawn/cuttlefish/crabs | | 1 medium |  | |  |  |  |
| **Code**  No | | **Food Type** |  |  |  |  |  |  | | --- | --- | --- | --- | --- | --- | --- | | | **Portion size** | **No of units taken at a time** | | **Per**  **Day** | **Per**  **Week** | **Per**  **Month** |
| **500** | **Fruits** | | | | | | | |
| **501** | Banana | | 1 medium |  | |  |  |  |
| **502** | Papaya | | 1 piece |  | |  |  |  |
| **503** | Apple | | 1 medium |  | |  |  |  |
| **504** | Mango | | 1 medium |  | |  |  |  |
| **505** | Orange | | 1 medium |  | |  |  |  |
| **506** | Pineapple | | Medium size |  | |  |  |  |
| **507** | Guava | | 1 medium |  | |  |  |  |
| **508** | Fruit salad | | 1 cup |  | |  |  |  |
| **509** | Dried fruits-Dates/ Resins | | 1 handful |  | |  |  |  |
| **600** | **Beverages** | | | | | | | |
| **601** | Nestamalt /Viva/Milo | | 1 glass |  | |  |  |  |
| **602** | Full cream milk | | 1 glass |  | |  |  |  |
| **603** | Non fat milk | | 1 glass |  | |  |  |  |
| **604** | Fresh milk | | 1 glass |  | |  |  |  |
| **605** | Plain tea | | 1 cup |  | |  |  |  |
| **606** | Coffee | | 1 cup |  | |  |  |  |
| **607** | Fizzy drinks | | 200 ml |  | |  |  |  |
| **608** | Cordial/Smack/Colman | | 1 glass |  | |  |  |  |
| **609** | Kola kada/rice kanji | | 1 glass |  | |  |  |  |
| **700** | **Miscellaneous** | | | | | | | |
| **701** | Yoghurt | | 1 cup |  | |  |  |  |
| **702** | Ice cream | | 1 cup |  | |  |  |  |
| **703** | Curd | | 1 cup |  | |  |  |  |
| **704** | Cheese | | 1 wedge |  | |  |  |  |
| **705** | Un-sweeten Biscuits | | 1 |  | |  |  |  |
| **706** | Cream/Sweet biscuits | | 1 |  | |  |  |  |
| **707** | Cake | | 1 medium size |  | |  |  |  |
| **Code**  No | | **Food Type** |  |  |  |  |  |  | | --- | --- | --- | --- | --- | --- | --- | | | **Portion size** | **No of units taken at a time** | | **Per**  **Day** | **Per**  **Week** | **Per**  **Month** |
| **708** | Watalappam/Pudding | | 1 cup |  | |  |  |  |
| **709** | Sweets | | 1 medium |  | |  |  |  |
| **710** | Sugar | | 1 tea spoon |  | |  |  |  |
| **711** | Toffee | | 1 |  | |  |  |  |
| **712** | Chocolate | | 1 |  | |  |  |  |
| **713** | Butter/Margarine | | 1 tea spoon |  | |  |  |  |
| **714** | Jam | | 1 tea spoon |  | |  |  |  |
| **715** | Tomato sauce | | 1 table spoon |  | |  |  |  |
| **716** | Mixtures/salted nuts | | 1 table spoon |  | |  |  |  |
| **800** | **Alcohol** | | | | | | | |
| **801** | Spirit | | 25 ml |  | |  |  |  |
| **802** | Beer | | 1 pint |  | |  |  |  |

1. **VISUAL ANALOGUE SCALES (VAS)**

- How **HUNGRY** do you feel *at this moment*?

**Not at all Very**

**HUNGRY HUNGRY**

- How **FULL** do you feel *at this moment*?

**Not at all Very**

**FULL FULL**

- How **STRONG** is your desire to eat *at this moment*?

**Not at all Very**

**STRONG STRONG**

- How **MUCH FOOD** do you think you could eat *at this moment*?

**None A large Amount**

**Investigator’s Signature:__________________________ Date:________________**

1. **COMPLIANCE**

**Total number of medicine/tablets expected to be used in last 30days :________**

**Total number of medicine/tablets actually used last 30 days :________**

**Total number of medicine /tablets missed/unused in last 30 days :________**

1. **ANALYSIS OF ADVERSE EVENT**

**Adverse events: Yes No**

**If yes, please specify**

| **Sr no** | **Adverse Effect (AE)** | **Severity*** | **Causality @** | **Drug responsible for AE** |
| --- | --- | --- | --- | --- |
|  |  |  |  |  |
|  |  |  |  |  |
|  |  |  |  |  |
|  |  |  |  |  |
|  |  |  |  |  |
| *** - Mild, Moderate, Severe**  **@ - Related (R), Possibly Related(PR), Definitely Related(DR) and Unlikely to be related (UR)** | | | | |

**Any serious adverse event (SAE): Yes No**

***If yes, please fill the SAE form enclosed at the end of the case report form and send to sponsor immediately.***

**Details of SAE:**

**_________________________________________________________________________________________________________________________________________________________________________________________________________________________________**

**Did the patient discontinue the study for any reason? Yes No**

**If yes, give reason:___________________________________________________________**

**Any missed doses: Yes No**

**Whether modification of test/reference drug is required? Yes No**

**Investigator’s Signature :___________________________ Date:________________**

1. **ANALYSIS OF ADVERSE EVENT**
2. **Patient’s Assessment of the Efficacy of Treatment**

Very Good Good Fair Unchanged

1. **Patient’s Assessment of the Tolerability of Treatment**

Very Good Good Fair Unchanged

1. **Physician’s Assessment of the Efficacy of Treatment**

Very Good Good Fair Unchanged

1. **Physician’s Assessment of the Tolerability of Treatment**

Very Good Good Fair Unchanged

**INVESTIGATOR’S DECLARATION**

**I CERTIFY THAT THE ABOVE DATA HAVE BEEN RECORDED BY ME/ UNDER MY SUPERVISION, HAVE BEEN VERIFIED AND ARE ACCURATE.**

_______________________________________ _____________________

**INVESTGATOR’S SIGNATURE AND SEAL DATE**

**…………………………………………………………………………………………………**

*For* *sponsor’s use only*

Are the CRFs filled up completely? Yes No

Is the diary card filled up completely? Yes No

_______________________________ _____________________

MONITOR’S SIGNATURE DATE

***Appendix X***

**Serious Adverse Event Reporting Form**

**Serious Adverse Events Reporting Form**

1. Patient Details

Initial & other relevant identifier (Hospital/ OPD record number)*___________________ Gender___________ Age________ Weight______ Height_________

1. Suspected Drug(s)

Generic name of the drug*__________________________________________________

Indication(s) for which suspect drug was prescribed or tested_______________________

Dosage form and strength___________________________________________________

Daily dose and regimen (specify units-eg, mg, ml, mg/kg)________________________

Route of administration_____________________________________________________

Starting date and time of day_________________________________________________

Stopping date and time, or duration of treatment_________________________________

1. Other Treatment(s)

Provide the same information for concomitant drugs (including non prescription/ OTC drugs) and non-drug therapies, as for the suspected drug(s).

Generic name of the drug*__________________________________________________

Indication(s) for which suspect drug was prescribed or tested_______________________

Dosage form and strength___________________________________________________

Daily dose and regimen (specify units-eg, mg, ml, mg/kg)________________________

Route of administration_____________________________________________________

Starting date and time of day_________________________________________________

Stopping date and time, or duration of treatment_________________________________

1. Details of Suspected Adverse Drug Reaction(s)

Full description of reaction(s) including body site and severity, as well as the criterion (or criteria) for regarding the report as serious. In addition to a description of the reported signs and symptoms, whenever possible, describe a specific diagnosis for the reaction.*

Start date (and time) of onset of reaction_______________________________________

Stop date (and time) or duration of reaction_____________________________________

De-challenge and re-challenge information _____________________________________

________________________________________________________________________

Setting (e.g., hospital, out-patient clinic, home, nursing home)

________________________________________________________________________________________________________________________________________________

1. Outcome

Information on recovery and any sequelae; results of specific tests and/or treatment that may have been conducted.

________________________________________________________________________________________________________________________________________________

For a fatal outcome, cause of death and a comment on its possible relationship to the suspected reaction; Any post-mortem findings.

________________________________________________________________________________________________________________________________________________

Other information: anything relevant to facilitate assessment of the case, such as medical history including allergy, drug or alcohol abuse; family history; finding from special investigations etc.

________________________________________________________________________________________________________________________________________________

1. Details about the Investigator*

Name:__________________________________________________________________

Address: ________________________________________________________________

________________________________________________________________________Telephone number:________________________________________________________

Profession (specialty):______________________________________________________

Date of reporting the event to Licensing Authority:_______________________________

Date of reporting the event to Ethics Committee overseeing the site__________________

Signature of the investigator

*Note: Information marked* must be provided*
